# Evaluating the impact of the 2010 Swedish choice reform in primary health care on avoidable hospitalization and socioeconomic inequities: an interrupted time series analysis using register data

**DOI:** 10.1186/s12913-024-11434-w

**Published:** 2024-08-23

**Authors:** Per E. Gustafsson, Osvaldo Fonseca-Rodríguez, Miguel San Sebastián, Bo Burström, Paola A. Mosquera

**Affiliations:** 1 Department of Epidemiology and Global Health, Umeå University, Umeå, 901 87 Sweden; 2 Department of Global Public Health, Karolinska Institutet, Stockholm, Sweden

**Keywords:** Primary health care, Health reform, Health inequities, Health care performance, Interrupted time series, Register research, Epidemiology, Sweden

## Abstract

**Background:**

The Swedish Primary Health Care (PHC) system has, like in other European countries, undergone a gradual transition towards marketization and privatization, most distinctly through a 2010 choice reform. The reform led to an overall but regionally heterogenous expansion of private PHC providers in Sweden, and with evidence also pointing to possible inequities in various aspects of PHC provision. Evidence on the reform’s impact on population-level primary health care performance and equity in performance remains scarce. The present study therefore aimed to examine whether the increase in private provision after the reform impacted on population-average rates of avoidable hospitalizations, as well as on corresponding socioeconomic inequities.

**Methods:**

This register-based study used a multiple-group interrupted time-series design for the study period 2001–2017, with the study population (*N* = 51 million observations) randomly drawn from the total Swedish population aged 18–85 years. High, medium, and low implementing comparison groups were classified by tertiles of increase in private PHC providers after the reform. PHC performance was measured by avoidable hospitalizations, and socioeconomic position by education and income. Interrupted time series analysis based on individual-level data was used to estimate the reform impact on avoidable hospitalization risk, and on inequities through the Relative Index of Inequality (RII).

**Results:**

All three comparisons groups displayed decreasing risk of avoidable hospitalizations but increasing socioeconomic inequities across the study period. Compared to regions with little change in provision after the reform, regions with large increase in private provision saw a steeper decrease in avoidable hospitalizations after the reform (relative risk (95%): 1.6% (1.1; 2.1)), but at the same time steeper increase in inequities (by education: 2.0% (0.1%; 4.0); by income: 2.2% (-0.1; 4.3)).

**Conclusions:**

The study suggests that the increase in private health care centers, enabled by the choice reform, contributed to a small improvement when it comes to overall PHC performance, but simultaneously to increased socioeconomic inequities in PHC performance. This duality in the impact of the Swedish reform also reflects the arguments in the European health policy debate on patient choice PHC models, with hopes of improved performance but fears of increased inequities.

## Background

An increasingly common direction in European health policy discourse centers on patient empowerment through market competition between healthcare providers [1]. As part of this development, multiple countries have transitioned towards patient choice models within primary healthcare (PHC), coupled with an increased dominance of private over public healthcare provision, e.g., several Nordic countries [2], the UK [3], and the Netherlands [4]. In other countries, like Finland, choice reforms have long been on the policy agenda, but not implemented [5]. This broader development has also been met with concerns about the health system and population consequences, not the least when it comes to equity in PHC provision [2, 3, 6]. Equitable PHC systems play a fundamental role for achieving equitable population health [7], and the ability of market forces to accomplish equitable provision have been questioned for decades, e.g., due to the risk of providers selecting patients with less care needs, or inequitable preconditions for patients making informed choices [8, 9]. A classical expression of this concern is Julian Tudor Hart’s 1971 ‘Inverse care law’, stating: ‘The availability of good medical care tends to vary inversely with the need for it in the population served. This inverse care law operates more completely where medical care is most exposed to market forces, and less so where such exposure is reduced. The market distribution of medical care is a primitive and historically outdated social form, and any return to it would further exaggerate the maldistribution of medical resources’ [10].

A Swedish 2010 PHC choice reform that opened for privatization and marketisation of Swedish PHC may be seen as an example of increased exposure to market forces, the impact of which may also be of interest to an international audience. The present study therefore seeks to contribute to our understanding of the consequences of privatization of PHC for performance and equity, with point of departure in the case of Sweden.

The responsibility for Swedish healthcare is decentralized to the 21 regions, which also collect taxes and deliver healthcare services. Sweden has gradually has increased its market-orientation across a range of welfare services since the 1990s [11], which contrasts with the country’s long tradition of publicly financed and provided healthcare [12, 13]. Marketization and privatization of Swedish welfare services have been particularly pronounced for PHC, which culminated in a 2010 choice reform in PHC [13–15]. While certain regions had implemented patient choice models before the 2010 reform, the 2010 reform enabled private PHC providers across the entire country to establish healthcare centers at any location, with patients choosing providers, and reimbursement from the region following the patient. New establishment of private PHC providers was seen as a way to increase total PHC provision nationally, as well as creating more options for the patients [16]. The increased competition between healthcare centers, driven by patients’ choice of provider, was intended to ultimately strengthen the patients’ status and improve care efficiency [12].

The reform was followed by a 20% net increase of the total number of health care centers in Sweden, concentrated to the first few years after the reform [17] and almost exclusively due to establishments of new private health care centers operating for profit, rather than public ones [18]. To illustrate, the share of private PHC providers increased from 28% in 2009, to more than 40% in 2013 [13, 18], but have remained fairly stable since then [17]. The overall increase in PHC provision after the reform thus entailed an overall transition of the Swedish PHC system towards increased emphasis on private rather than public provision. However, the number of new establishments varied markedly across the 21 regions of Sweden [13]. Specifically, while certain regions experienced a markedly increased share of private PHC centers, other regions saw a more moderate or little change at all; either because of less attractive markets (e.g., rural regions, such as Jämtland/Härjedalen), or due to regional policies having enabled a dominance of private providers already before the national reform (e.g., Stockholm and Halland). As such, the increase in private PHC provision that was specifically initiated by the reform was very unequally distributed across Sweden.

The diverse regional expressions of the reform when it comes to PHC provision illustrates the mentioned concerns about how choice reforms may affect equity in PHC provision [3, 6], and was an issue debated within Sweden before the reform was introduced [12, 14], and still remains [19]. Equity is a core principle in Swedish health policy, guided by explicit equity goals in public health policy [20] and with legislation stating that the goal of healthcare is good health and healthcare on equal terms for the entire population [21]. Equity in healthcare is in this context thus understood as healthcare on equal terms for the entire population [21], with inequity in healthcare representing systematic differences (inequalities) in healthcare between population groups that are unfair and avoidable. Despite this strong status of equity in the Swedish policy landscape, it was not an issue that was comprehensively considered by policymakers when outlining the 2010 reform [22].

Various expressions of inequitable consequences have indeed been reported after the Swedish choice reform [8, 19]. For example, new private health care centers have been concentrated to affluent and urban areas rather than according to the needs of the population [18], and with private health care centers attracting socioeconomically well-off patient groups [23]. While overall PHC utilization increased overall after the reform [24–26], certain evidence suggests that utilization increased disproportionally among socioeconomically privileged groups [24, 26, 27], and it is unclear to whether the overall increased utilization was coupled with a change in care quality [25]. These pieces of evidence exemplify the challenges of detailing the complex impacts of a reform with a wide web of potential downstream consequences for the population served.

One commonly used proxy for overall PHC performance is hospitalizations due to ambulatory care sensitive conditions (ACSC), or avoidable hospitalizations. This indicator includes causes of hospitalizations that are potentially preventable by the PHC system [28, 29] and taps into multiple aspects of PHC performance, such as continuity [30, 31], accessibility [28, 32], and resources [28, 31]. The long-term trend of ACSC hospitalizations in Sweden has declined from 2009 to 2018, however with large regional variations [33]. There is also a socioeconomic gradient in the rate of ACSC hospitalizations, as showed in a recent comparison of ACSC hospitalizations of the capitals of Denmark, Finland and Sweden across 2000–2015 [34]. This study reported a gradual decrease in ACSC hospitalization rates in Stockholm over the study period, albeit stagnating among the oldest population groups, and with stable income-related, and reduced small-area geographical, inequities in ACSC. The study did not specifically examine the impact of the 2010 choice reform, however, and relevant examples from the literature are far sparse. Two studies have reported little effect of the choice reform on overall avoidable hospitalizations [35, 36], but with worse outcomes in regions with long-term dominance of private providers, and uncertain influence of the degree of new (private) health care centers [36]. These studies were however based on aggregated data which precludes consideration of individual-level confounders, and neither examined the impact on equity in performance. One recent study has reported overall increased complex inequities in ACSC in Sweden following the reform [37], but was unable to attribute this development specifically to features of the reform, due to the lack of a comparative design.

Taken together, while the Swedish choice reform in PHC has contributed to an overall but heterogenous increase in private health care centers and utilization in Sweden, its impact on PHC performance and equity in performance are still, after almost 15 years since its introduction, uncertain [19]. One the one hand, the overall expansion of private health care centers might be hypothesized to reduce the overall rate of ACSC, through increased overall access to PHC. One the other hand, if this increased access is inequitably distributed, it might simultaneously contribute to an increase of the socioeconomic inequities in rates of ACSC. The aims of the present study were therefore to examine whether a key expression of the choice reform, the increase in private PHC provision after the reform, impacted on population-average rates of avoidable hospitalizations, as well as on education- and income-related relative inequities in hospitalizations, comparing regions with small, moderate and large increase in private PHC provision after the reform. While we acknowledge that ‘inequities’ and ‘inequalities’ are used heterogeneously in the literature [38], in this report, we will use the term ‘inequities’ to refer to differences between socioeconomic groups, as we consider systematic differences in PHC performance between these socioeconomic groups to be both unfair and avoidable.

## Methods

### Study design

This study used a multiple-group interrupted time-series (ITS) design based on individual-level data. The ITS design is a quasi-experimental evaluation design that is considered the strongest option for causal inference when randomization of subjects is not an option, and is particularly suitable for evaluation of population-level changes that occur at a specific time point, such as health reforms, if time series data is available [39, 40]. The design takes into account underlying (e.g., secular) trends of the outcome, which are not attributed to the intervention and therefore risk introducing bias the effect estimates. This is done by comparison of trends of the outcome during a period after the intervention to corresponding trends period before the intervention (rather than single observations before and after the intervention). The ITS design is commonly conducted within a single population (single-group, or uncontrolled, ITS), but can be extended to incorporate a comparison group (multiple-group, or controlled, ITS) [41, 42], which provides further control of bias from competing interventions or events occurring close to the intervention under study. The most common application is on aggregated (e.g., country-level) data, but it can also be conducted on individual-level data [42], which enables control for individual-level time-varying confounders.

The study period was 2001–2017. The choice reform was introduced nationally on Jan 1st, 2010, and the study period was divided into pre-reform period (2001–2009; 10 yearly observations), and a post-reform period (2010–2017, 8 yearly observations). Three comparison groups were constructed to capture region-level increase in private PHC provision occurring after the choice reform, following the categorization of our previous report [36]. The classification was based on public statistics from the Swedish Association of Local Authorities and Regions on the number of public and private health care centers per region and year 2009–2016.While the reform was introduced nationally in 2010, the categorization into comparison groups capitalizes on between-region heterogeneity when it comes to the establishment of new private health centers, as a central indicator of the de facto implementation of the reform. First, the proportion of private health care centers (private/total) were calculated for each region, in the year before the reform (2009 used as a baseline) and the years following the introduction of the reform (averaged across 2010–2016) [13]. Second, all regions were ranked by the absolute and relative change in proportion of private providers from before to after the reform. Lastly, the regions were classified according to tertiles, in order to achieve balanced comparison groups, and thereby minimize the risk for individual regions disproportionally affecting the group estimates. The seven regions with greatest increase in the proportion of private health care centers (> 10% absolute increase and > 60% relative increase) were categorized as *high* (regions of Uppsala, Södermanland, Jönköping, Kronoberg, Västra Götaland, Värmland, Dalarna); the seven counties with the smallest increase (or decrease) in the proportion (< 6% absolute increase and < 15% relative increase) was categorized as *low* (regions of Kalmar, Gotland, Blekinge, Halland, Örebro, Västmanland, Jämtland); and seven regions comprising the middle tertile were categorized as *moderate* increase of privatization (Stockholm, Östergötland, Skåne, Gävleborg, Västernorrland, Västerbotten, Norrbotten). The low group was used as the reference group in all analyses.

### Study population and data

The study population included all residents in Sweden aged 18–85 years each year 2001–2017, in total comprising *N* = 125,438,725 observations. To facilitate the computational challenges with such large data, a random sample of 1 million individuals were drawn annually from each of the three comparison groups, resulting in an analytical sample of 51,000,000 observations uniformly distributed across three comparison groups and the 17 study years (2001–2017).

Individual-level data on the study population was retrieved for each year over the study period. The data sources were multiple registers with total population coverage, managed by Swedish public authorities. Data on hospitalizations was retrieved from the National inpatient register of The National Board of Health and Welfare, and all socioeconomic and demographic information from the Longitudinal integrated database for health insurance and labour market studies (LISA) of Statistics Sweden. All data was individually linked by the unique Swedish Personal Identity Number.

### Variables

#### Performance outcome and socioeconomic indicators

The outcome variable comprised *avoidable hospitalizations*, corresponding to hospitalization due to ambulatory care sensitive conditions (ACSC), following the classification of ACSC diagnoses by The National Board of Health and Welfare [43, 44]. It was operationalized as a binary variable per year (0 = no ACSC hospitalizations; 1 = at least one ACSC hospitalization).

To estimate the bivariate phenomenon of socioeconomic inequities in ACSC hospitalizations, the ACSC hospitalization outcome was used in combination with two complementary socioeconomic indicators (procedures described in Statistical analysis). *Education* was based on the highest formal educational degree and classified into five levels according to Statistics Sweden’s classification SUN2000 [45] (no or basic education; primary education; secondary education; basic tertiary education (less than three years); advanced tertiary education (three years or more)). *Income* was based on disposable annual household income weighted by family composition and was divided into quintiles of the annual income distribution (quintile 1 = poorest to quintile 5 = richest).

#### Potential confounders

Several covariates were operationalized in order to further control for potential confounding. First, as regional patient choice models were implemented ahead of the 2010 national reform in eight regions (Halland, Västmanland, Stockholm, Uppsala, Kronoberg, Skåne, Östergötland, Västra Götaland [46]) and this could potentially influence the subsequent impact of the 2010 national reform, a variable indicating *Early implementation* was created by grouping region of residence by timing of implementation (0 = region implemented 2010, 1 = region implemented before 2010). Three early implementation regions each belonged to the high and mid implementation comparison groups, with two in the low implementation comparison group.

Second, as the ITS design relies on comparison over time and geographical regions, demographic developments and composition of the regional populations could potentially confound the results. *Age* was measured in years and grouped into young adulthood (18–35 years), mid-adulthood (36–64 years), and old adulthood (65–85 years); *Gender* as indicated by legal sex (woman or man); and *Country of birth* coded as Nordic countries, other high-income country (HIC), or Low- or middle-income country (LMIC). The above variables were considered as potential confounders for all analyses.

Additionally, the following confounders were identified for analyses considering population-average ACSC risk, but were not included in the analyses of inequities in ACSC to avoid the risk of overadjustment for potential mediators. *Labor market position* was based on the main source of income each year, with ten categories: employed; studying; care of child/close one; sickness benefits; unemployed; early retirement; social benefits; labor market program; age retirement; and no income. As a measure of *Urbanicity*, municipality of residence was classified into rural, mixed urban/rural, and urban [47]. *Education* and *income*, operationalized as above, were also included as covariates in the analyses of ACSC hospitalization rates only.

### Statistical analysis

Descriptive statistics are reported as percentages (%). Intermediate analyses comprised estimation of ACSC hospitalizations rates as well as education- and income-related inequities in ACSC hospitalizations, by period (pre-reform and post-reform collapsed within period) and by comparison group (low, moderate, and high implementation). All analyses were performed on the individual-level sample of 51,000,000 individuals.

For the main analyses, we conducted a series of multiple-group interrupted time series analyses (ITSA) based on individual-level data [42], using generalized linear model (glm) with binomial family and log link for estimation of relative risks. In all analyses, ACSC hospitalizations was used as the dependent variable, and the low implementation group was used as the reference, with the moderate and high groups as intervention groups.

To examine the reform impact on population-average avoidable hospitalizations (Aim 1) a multiple-group ITSA with ACSC hospitalizations as the outcome was run. The glm model was as follows:

1 $$Y_t\:=\:\beta_0\;+\:\beta_1T_t\;+\:\beta_2X_t\;+\:\beta_3Z\:+\:\beta_4TtXt\:+\:\beta_5TtZ\:+\:\beta_6XtZ\:+\:\beta_7TtXtZ\;+\;\varepsilon_t$$

where Y_t_ is the *outcome* (annual ACSC hospitalizations), T represents *time* (year, 0 = 2001, 1 = 2002 … 17 = 2017), X represents *period* (0 = pre-reform period 2001–2009; 1 = post-reform period 2010–2017); and Z refers to a dummy variable with three categories denoting the *comparison group* (0 = Z_0_ = reference group, omitted; 1 = Z_1_ = middle group; 2 = Z_2_ = high group), and their interaction effects. Here, the coefficient for *TtXtZ*_*1*_ (mid vs. low) and *TtXtZ*_*2*_ (high vs. low) effects, i.e., the Time*Group*Period effects, are of main interest. The corresponding estimate (*β*_*7*_) tests whether the slope of the post-reform trends in ACSC hospitalizations differed between the comparison groups, taking into account the corresponding pre-reform trends, and thus represents the impact of the reform on ACSC trends. Two models were run, one crude (Model 1, shown in Eq. 1, above) as well as one model additionally adjusted for age, sex, country of birth, early implementation, labor market position, urbanicity, education, income (Model 2).

To examine the reform impact on inequities in ACSC hospitalizations (Aim 2), we performed a novel extension of the ITSA. To quantify the magnitude of inequities, we estimated the ‘Relative Index of Inequality’ (RII), which is a standardized measure of relative inequalities capturing the social gradient in an outcome [48, 49]. Note that while the present study focuses on inequities (inequalities that are avoidable and unfair), we will refer to the measure as ‘Relative Index of Inequality’, as that is its most common designation. The RII can be interpreted as the relative risk moving from the theoretically most favorable social position (0) to the most disadvantaged position (1). The relative size of the social categories is taken into account by first transforming the socioeconomic indicators into a *ridit score*, which uses the mid-point of the cumulative proportion of each socioeconomic category along educational level and income quintile, respectively [49]. The ridit scores were reverse coded so that a higher RII indicates a steeper social gradient in health (larger magnitude of the health inequality or inequity).

Interrupted time series analyses are conventionally used to estimate population-average impact on an outcome, as done for the first aim (Eq. 1), rather than to estimate the impact on inequities. To enable estimation of the RII within the ITS framework, we used the fact that the RII can be estimated as a relative risk in regression models. We extended the basic ITSA model (Eq. 1) to also incorporate ridit main and all interaction effects, with separate models for education and income, respectively. Specifically, we extended the ITSA model (Eq. 1) by adding the ridit as a main effect, as well as all 2-, 3- and 4-way interaction effects, as per the following model:

2 $$\begin{array}{c}Y_t\:=\:\beta_0\:+\:\beta_1T_t+\:\beta_2X_t\:+\:\beta_3Z\:+\:\beta_4ridit_t\:+\:\beta_5TtXt\:+\:\beta_6TtZ\:+\:\beta_7XtZ\:+\:\beta_8T_t\times ridit_t+\\\beta_9X_t\times ridit_t\;+\;\beta_{10}Z\times ridit_t\;+\;\beta_{11}TtXt\times ridit_t\;+\;\beta_{12}TtZ\times ridit_t\;+\;\beta_{13}XtZ\times ridit_t\;+\\\beta_{14}TtXtZ\times ridit_t\;+\;\varepsilon_t\end{array}$$

This extended model (Eq. 2) thus uses the same Y_t_ (annual ACSC hospitalizations) and contains all effects included in the model for the first aim (Eq. 1), but with the addition of ridit main and interaction effects. In this model, all effects (*β*s) that include the ridit can be interpreted analogously to the corresponding ITS effects without the ridit term, but instead reflecting the relative change of RII rather than of the risk of the outcome itself. As an example, and most importantly, the coefficient for the 4-way interaction term *TtXtZ×ridit*_*t*_ is of main interest as it represents whether the slope of the post-reform trends in RII differed between the mid and low comparison groups (*β*_*14*_*TtXtZ*_*1*_*)*, and high and low comparison groups (*β*_*14*_*TtXtZ*_*2*_*)*, accounting for the pre-reform trends. This estimate thus reflects the impact of the reform on the trends in inequities in avoidable hospitalizations. Two models were run for education- and income-related, respectively, including a crude model (Model 1, shown in Eq. 2), above), and a model additionally adjusted for age, sex, country of birth, and early implementation (Model 2).

As a recent study has reported that the trends of ACSC in Stockholm, Sweden, have developed in a less favorable direction for older adults [34], auxiliary analyses comprised rerunning all analyses specifically for the oldest age group (aged 65–85 years). The inferences from these auxiliary analyses were however identical to the analyses on the total sample (data available on request).

## Results

Descriptive statistics over the entire study period showed a slightly lower occurrence of ACSC hospitalizations in mid-implementing regions (Table 1), i.e., the group of regions with a mid-tertile increase in share of private PHC centers after the reform. The low- and high-implementing regions were more similar to each other when it comes to ACSC risks. Regarding the demographic and socioeconomic distribution, the mid-implementing regions had a considerably greater proportion of the population living in urban municipalities, as well as highly educated, high income, and Nordic born groups, compared to the low- and high implementers. The low-implementing group had an older population, and a dominance of rural or mixed urban/rural municipalities, compared to the middle-and high implementing regions.

**Table 1:** Characteristics of the study population

Variable	Total sample	Comparison group		
		Low	Middle	High
**Number of observations**	51,000,000	17,000,000	17,000,000	17,000,000
**ACSC hospitalizations**	1.12	1.16	1.08	1.13
**Education**				
Advanced tertiary	17.13	14.33	20.53	16.51
Basic tertiary	12.98	12.29	13.94	12.70
Secondary	40.60	41.96	39.20	40.63
Primary	16.46	16.83	15.90	16.65
No or basic	12.84	14.59	10.42	13.51
**Income (quintiles)**				
Highest	19.30	17.02	22.72	18.16
High	20.29	20.20	20.13	20.56
Medium	20.62	21.50	19.38	20.97
Low	20.71	22.19	18.85	21.08
Lowest	19.09	19.10	18.93	19.23
**Sex**				
Woman	50.47	50.35	50.69	50.37
Man	49.53	49.65	49.31	49.63
**Age**				
Young	28.24	26.39	29.74	28.60
Mid	48.46	48.48	48.73	48.17
Old	23.30	25.13	21.53	23.23
**Place of birth**				
Nordic countries	88.90	91.52	86.03	89.15
Other high income areas	5.36	4.43	6.47	5.17
Other regions	5.74	4.05	7.50	5.68
**Labor market position**				
Employed	59.05	57.91	59.92	59.32
Studying	3.92	3.54	4.29	3.92
Care of child	1.51	1.44	1.59	1.51
Sick	1.56	1.59	1.52	1.55
Unemployed	1.19	1.23	1.21	1.13
Early retired	4.57	4.69	4.36	4.65
Social benefits	1.64	1.50	1.70	1.72
Labour market program	1.12	1.21	1.09	1.05
Age retirement	23.43	25.30	21.73	23.26
Lacks income	2.03	1.60	2.60	1.90
**Urbanicity**				
Rural	20.03	26.17	12.51	21.41
Mixed	57.08	73.83	38.69	58.73
Urban	22.89	0.00	48.81	19.86

Descriptive statistics of the total study population and by groups of low, middle and high implementers of the 2010 Swedish Choice in Primary Health Care reform. Numbers are percentages (%)

**Fig. 1:**
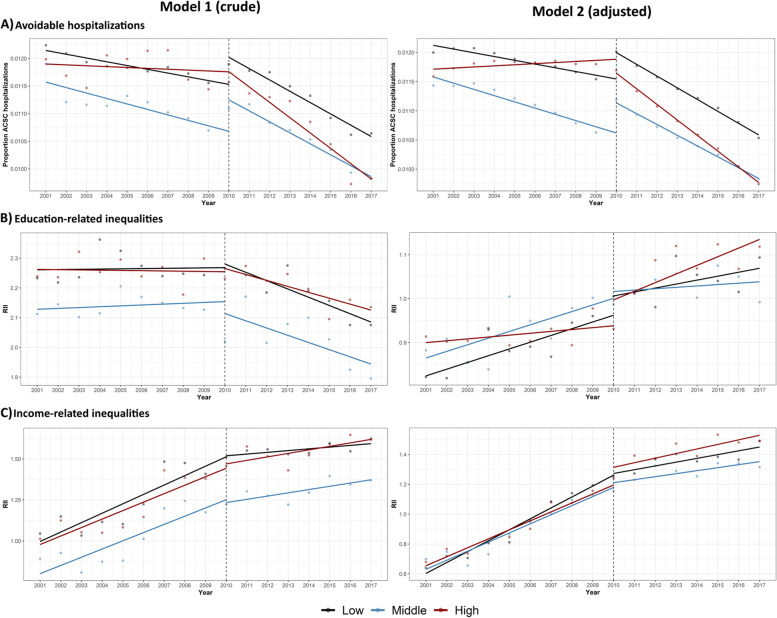
Trends of avoidable hospitalizations and socioeconomic inequities in avoidable hospitalizations before and after choice reform. Illustration of interrupted time series analyses examining the trends of ACSC hospitalizations (Panels **A**), education-related inequities in ACSC hospitalizations (Panels **B**), and income-related inequities in hospitalizations (Panels **C**), respectively, before and after a 2010 health reform in Sweden, in crude model (Model 1) and model adjusted for Age, Gender, Country of birth, Labor market position, Urbanicity, Income, Education and Early Implementation (avoidable hospitalizations); Age, Gender, Country of Birth and Early Implementation (education- and income-related inequities) (Model 2)

When it comes to the population-average ACSC before and after the reform (Table 2), the risk of hospitalization was found to be higher during the pre-reform compared to the post-reform period, with the same pattern in the total population as well as in each of the three comparison groups. This descriptive pattern was further confirmed after consideration of the pre- and post-reform trends in ACSC hospitalizations, by ITS analysis (Table 3; Fig. 1, Panel A). Crude ITS analyses (Model 1) revealed a slight downward trend already in the pre-reform period, with an even steeper decline in the postreform period, in all three comparisons groups. In adjusted analyses taking covariates into account, suggested disparate developments for the three comparison groups (Fig. 1). In the low- and middle-implementing regions, ACSC hospitalizations decreased throughout the study period, with only slightly steeper decrease in the trends after the reform. The high-implementing regions instead displayed slightly increasing trends before the reform, with a sharper decrease after the reform. The change in trends from before to after the reform was confirmed to be of larger magnitude in the high- compared to the low-implementing regions in the ITS analysis (Table 4, Time*Group*Period effect, RR(95%CI) = 0.984 (0.979; 0.989)). This estimate corresponds to a 1.6% sharper decline in ACSC hospitalizations after the reform in the high- compared to the low-implementing regions, taking into account the respective pre-reform trends. Visual inspection of the adjusted model in Fig. 1 thus suggests that this result was largely explained by steeper pre-reform upward trends in the high-implementing regions, more than by a steeper downward trend after the reform.

**Table 2:** Avoidable hospitalizations and socioeconomic inequities in avoidable hospitalizations before and after choice reform

Outcome and group	Study period	
	Pre-reform (2001-09)	Post-Reform (2010-17)
**ACSC hospitalizations**		
Total sample	1.16 (1.16–1.17)	1.09 (1.08–1.09)
Low implementers	1.19 (1.18–1.19)	1.13 (1.12–1.14)
Middle implementers	1.12 (1.111–1.12)	1.06 (1.05–1.06)
High implementers	1.18 (1.18–1.19)	1.08 (1.07–1.09)
**RII by education**		
Total sample	2.22 (2.20–2.23)	2.13 (2.12–2.15)
Low implementers	2.26 (2.24–2.29)	2.19 (2.16–2.21)
Middle implementers	2.14 (2.12–2.16)	2.03 (2.01–2.06)
High implementers	2.26(2.24–2.28)	2.20 (2.17–2.23)
**RII by income**		
Total sample	1.13 (1.12–1.15)	1.46 (1.45–1.48)
Low implementers	1.22 (1.20–1.25)	1.55 (1.53–1.58)
Middle implementers	1.00 (0.98–1.02)	1.30 (1.28–1.33)
High implementers	1.18 (1.16–1.21)	1.54 (1.52–1.57)

Absolute risk (%) of hospitalizations due to Ambulatory care sensitive conditions (ACSC) and Relative Index of Inequalities (RII) by education and income, by period (pre- and postreform) and comparison group (low, middle, and high implementers), with 95% confidence interval (CI)

**Table 3:** Estimated impact of choice reform on avoidable hospitalizations

Effect	Model 1 (crude)		Model 2 (adjusted)	
	RR (95% CI)	*p*	RR (95% CI)	*p*
Time (T)	0.994 (0.992; 0.997)	**< 0.001**	0.993 (0.991; 0.995)	**< 0.001**
Period (X)	1.043 (1.025; 1.062)	**< 0.001**	1.049 (1.031; 1.067)	**< 0.001**
Group (mid) (Z)	0.956 (0.938; 0.974)	**< 0.001**	1.081 (1.061; 1.101)	**< 0.001**
Group (high) (Z1)	0.975 (0.958; 0.993)	**0.008**	0.997 (0.979; 1.016)	0.773
Time*Period (XT)	0.988 (0.984; 0.991)	**< 0.001**	0.988 (0.985; 0.992)	**< 0.001**
Time*Group (mid) (ZT)	0.997 (0.994; 1.000)	0.066	0.999 (0.995; 1.002)	0.375
Time*Group (mid) (ZT1)	1.004 (1.001; 1.008)	**0.008**	1.009 (1.006; 1.013)	**< 0.001**
Period*Group (mid) (ZX)	1.010 (0.985; 1.036)	0.443	1.014 (0.989; 1.040)	0.268
Period*Group (high) (Z1X)	0.961 (0.937; 0.985)	**0.002**	0.944 (0.921; 0.967)	**< 0.001**
Time*Period*Group (mid) (TXZ)	1.002 (0.997; 1.008)	0.370	1.004 (0.999; 1.010)	0.112
Time*Period*Group (high) (TXZ1)	0.988 (0.983; 0.993)	**< 0.001**	0.984 (0.979; 0.989)	**< 0.001**

Estimates based on trends of avoidable (ACSC) hospitalizations, before and after a 2010 health reform in Sweden (*N* = 51,000,000), in unadjusted model (Model 1) and model adjusted for Age, Gender, Country of birth, Labor market position, Urbanicity, Income, Education, and Early implementation (Model 2). Estimates are risk ratios (RR) with 95% confidence interval (CI) and *p* value

**Table 4:** Estimated impact of choice reform on socioeconomic inequities in avoidable hospitalizations

Effect	Relative inequalities (RII) by Education				Relative inequalities (RII) by Income			
	Model 1 (crude)		Model 2 (adjusted)		Model 1 (crude)		Model 2 (adjusted)	
	RII (95% CI)	*p*	RII (95% CI)	*p*	RII (95% CI)	*p*	RII (95% CI)	*p*
Time (T)	1.001 (0.992; 1.011)	0.839	1.022 (1.013; 1.031)	**< 0.001**	1.059 (1.049; 1.068)	**< 0.001**	1.082 (1.072; 1.092)	**< 0.001**
Period (X)	1.010 (0.940; 1.086)	0.785	1.013 (0.949; 1.080)	0.705	1.008 (0.942; 1.079)	0.808	1.007 (0.939; 1.080)	0.839
Group (mid) (Z)	0.874 (0.812; 0.941)	**< 0.001**	1.104 (1.033; 1.180)	**0.004**	0.829 (0.774; 0.887)	**< 0.001**	1.109 (1.034; 1.189)	**0.004**
Group (high) (Z1)	1.003 (0.931; 1.081)	0.928	1.070 (1.000; 1.144)	**0.049**	0.988 (0.922; 1.059)	0.734	1.068 (0.995; 1.146)	0.069
Time*Period (XT)	0.972 (0.957; 0.987)	**< 0.001**	0.991 (0.978; 1.004)	0.183	0.954 (0.941; 0.968)	**< 0.001**	0.948 (0.934; 0.962)	**< 0.001**
Time*Group (mid) (ZT)	1.002 (0.989; 1.015)	0.758	0.999 (0.987; 1.011)	0.808	0.993 (0.981; 1.005)	0.231	0.983 (0.971; 0.996)	**0.009**
Time*Group (mid) (ZT1)	0.998 (0.985; 1.012)	0.794	0.991 (0.979; 1.003)	0.150	0.994 (0.982; 1.006)	0.341	0.987 (0.974; 0.999)	**0.039**
Period*Group (mid) (ZX)	0.948 (0.858; 1.048)	0.299	0.990 (0.905; 1.084)	0.836	0.978 (0.890; 1.074)	0.635	1.029 (0.934; 1.134)	0.564
Period*Group (high) (Z1X)	1.000 (0.904; 1.106)	0.994	1.040 (0.950; 1.139)	0.398	1.024 (0.931; 1.127)	0.620	1.120 (1.016; 1.236)	**0.023**
Time*Period* Group (mid) (TXZ)	1.002 (0.981; 1.023)	0.873	0.995 (0.977; 1.014)	0.626	1.017 (0.997; 1.037)	0.094	1.010 (0.990; 1.031)	0.328
Time*Period* Group (high) (TXZ1)	1.010 (0.988; 1.031)	0.379	1.020 (1.001; 1.040)	**0.042**	1.016 (0.996; 1.037)	0.112	1.022 (1.001; 1.043)	**0.040**

Summary of two multiple-group interrupted time series analyses examining trends of relative education- and income-related inequities in avoidable (ACSC) hospitalizations measured, before and after a 2010 health reform in Sweden (*N* = 51,000,000), in unadjusted model (Model 1) and model Age, Gender, Country of Birth, and Early implementation (Model 2). All reported effects represent an interaction term with the ridit score to estimate RII; for simplicity, the remaining main and interaction effects not containing the ridit are not shown in table, but available on request. Estimates are relative index of inequalities (RII) with 95% confidence interval (CI) and p value

The development of socioeconomic inequities during the pre- and post-reform periods (Table 2) suggested a more complicated pattern, which however was similar across the three comparison groups. First, the magnitude of relative inequities in ACSC hospitalizations were of considerably larger magnitude by education than by income, both in the pre- and post-reform period. Second, while education-related inequities were lower during the post- compared to the pre-reform period, income-related inequities were overall higher in the post- compared to pre-reform period.

Examining these descriptive inequity patterns by considering the trends in ITS analysis and covariates (Table 4; Fig. 1, Panel B for education, Panel C for income), more nuanced patterns emerged. Unadjusted analyses (Model 1) suggested that education-related inequities remained stable before the reform but declined after the reform, similarly in all three groups (Fig. 1). However, adjusted analyses (Model 2) instead showed increasing education-related inequities in ACSC hospitalizations both before and after the reform in all three groups. Moreover, while the low- and middle implementing groups showed a steeper trend before than after the reform, the high-implementing group displayed showed the reverse, with steeper trends in education-related inequities after the reform (Fig. 1). The corresponding group differences in pre-post change in trend (Time*Period*Group (high)) was estimated at 2.0% higher in high- compared to low-implementing regions (Table 4, RII (95%CI) = 1.020 (1.001; 1.040)). Income-related inequities displayed more similar developments in unadjusted (Model 1) and adjusted (Model 2) analyses. Inequities increased sharply before the reform, and continued increasing inequities at a slightly slower rate after the reform (Fig. 1). While this general pattern was seen in all groups, the high-implementing regions displayed a slightly steeper increase after the reform. This was expressed in the ITS analysis as a 2.2% slower decrease in inequities after the reform in the high-implementing compared to the low-implementing group (Table 4, Time*Period*Group (high), RII (95%CI) = 1.022 (1.001; 1.043)). Both education- and income-related inequities thus displayed a worse development in high-implementing than low-implementing regions after the reform. Inspection of adjusted models in Fig. 1 suggests that these results were underpinned by a combination of flatter pre-reform inequity increase, together with a steeper post-reform inequity increase, in the high-implementing regions.

## Discussion

This study illustrates population-level consequences of transition towards increased privatization and marketization of PHC, using the case of a 2010 Swedish choice reform. The study specifically examined how one central expression of the reform, the expansion of private PHC centers, impacted on population-average and socioeconomic inequities in an indicator of PHC performance. It did so by a novel analytical procedure estimating an established inequity measure, the Relative Index of Inequality, within a multiple-group ITS design. The results suggest that regions with a large increase in private provision experienced to larger reductions in ACSC hospitalization, compared to regions with little change in private provision. However, the same high-implementing regions displayed a worse development when it comes to socioeconomic inequities in ACSC hospitalizations. These findings are in general accordance with our hypotheses of the impact of the reform. However, as suggested by the large universal decrease in ACSC hospitalizations and increases in socioeconomic inequities in ACSC hospitalizations regardless of increase on private provision, other forces, beyond the choice reform, seem to have stronger influence on the societal trends in avoidable hospitalizations and inequities.

This is the first study using individual-level total population data and strong quasi-experimental design to examine the impact of the 2010 reform on PHC performance and inequities. The study thus provides a rigorous examination of the consequences of marketization and privatization of PHC for the case of Sweden. Through the ubiquity of patient choice models in Europe [1–4, 6], these findings should be informative for other contexts, including the crucial issue of how patient choice models may affect equity in PHC [1, 2, 4, 6, 8, 9, 12, 22, 50–52]. Additionally, the novel analytical procedure illustrated in this study is broadly applicable for public health evaluation of the equity impact of reforms, policies, or other large-scale public health interventions.

In interpreting the results of the present study, it is important to point out that private health care centers accounted for practically all new establishments after the reform. This means that it is impossible to disentangle an increase in private provision on the one hand, from that of a general increase in PHC provision and access on the other. The level of private provision indeed varied considerably between our comparison groups, with the high-implementing group comprising regions with both smaller and larger presence of private providers before the reform, and with certain regions implementing choice models ahead of the national reform. Our previous report has shown that regions which had not increased their share of private provision precisely because there already was a dominance of private PHC providers already before the reform comprise regions with a particularly poor development when it comes to ACSC hospitalization [36]. While previous Swedish research has however been inconclusive when it comes to whether PHC care quality indicators differ between public and private providers [53], this is complex to ascertain because of the selective population groups, with lesser care needs, among private providers [18, 23, 53].

In line with national figures [33], our results showed that all comparison groups displayed steeply decreasing trends in ACSC hospitalizations after the reform. This suggests that other factors, acting on the population level, have exerted a larger influence on the development of ACSC, than did the increase in (private) PHC provision enabled by the choice reform. Our findings of slightly more positive development in regions increasing their number, and share, of private providers, seemed to be dependent on stagnant or increasing ACSC rates before the reform, more so than markedly decreasing trends after the reform (see Fig. 1, adjusted model). Previous studies using aggregated data by municipality or region have been unable to show certain improvement in population-average avoidable hospitalizations after the reform [35, 36], but with point estimates in the same direction and of similar magnitude as in the present study. The present study thus provides robust evidence supporting that the increased provision initiated by the reform has improved population-average PHC performance nationally.

On the other hand, the fact that socioeconomic inequities in avoidable hospitalizations continued to rise after the reform suggests that the reform did not improve the equity in PHC in Sweden. Moreover, a particular source of concern is that results suggest increasing rather than decreasing inequities in ACSC hospitalizations among high-implementing regions, despite the increased overall PHC provision in the same regions. This could be rooted in the previously demonstrated increased inequities in PHC localization and utilization after the reform [24, 26, 27]. It should also be noted that recent studies have found that the inequity impact of the reform may follow more intricate patterns than is revealed by consideration of single socioeconomic dimensions. For example, heterogenous developments of socioeconomic inequities in GP visits by age and gender [26], and an increase particularly when it comes to complex, or multidimensional, inequity in ACSC hospitalizations, after the reform [37]. The location of new private PHC centers after the reform also varies within regions, for instance between urban and rural areas of the same region, which would contribute to the geographical inequity demonstrated in previous research [18]. Furthermore, multiple regions put strategies into place to counteract inequitable consequences of the reform, e.g., through regulations limiting risk selection of patients [54], which should be expected to reduce the inequitable impact of the reform. The present study thus likely underestimates the full extent of reconfiguration of PHC inequities in the wake of the reform.

Taken together, the consequence of the reform seems to illustrate a trade-off between improved overall PHC performance on the one hand, and equity in PHC performance on the other, with both impacts of comparable magnitude (1.6% for overall performance; 2.0-2.2% for inequities), and small in relation to the overall secular trends not attributed to the reform. As noted above, evidence does not inconclusively support different PHC quality in private compared to public health care centers [53], but poor PHC performance has been shown in regions with sustained dominance of private providers [36]. It is therefore possible to interpret our finding of improved overall performance in high-implementing regions as a consequence of an overall increase in provision enabled by the reform, rather than attributed to ownership of said providers. The overall increase in provision after the reform may, for example, have enabled certain regions with mounting challenges when it comes to PHC performance to better meet the overall care needs of the population, e.g., by the overall increased GP visits following the reform shown in previous research [24–26]. Despite the overall increase in provision, the precise localization and population groups served by private PHC centers are driven by profit rather than by healthcare needs of the population [18, 23, 53]. The finding that inequities increased after the reform despite the increase in provision, as seen in our study, could therefore potentially be explained by the market-orientated mode of provision promoted by the choice reform. These findings could be viewed as an expected consequence of the policy-makers explicit intentions with the reform, and also the lack of considerations when it comes to the potential impact on equity [22]. The demonstration of these impacts in the present study are however remarkable considering the central role the Swedish PHC system is intended to play when it comes to meeting the societal challenge of inequities in health [20, 21].

### Methodological considerations

The strengths of the presents study include a large dataset randomly sampled from the total population, high quality register data with practically complete coverage, a rigorous interrupted time series design, and a novel analytical procedure for estimating the inequity impact using this design. While the population only included adults up to 85 years, which limits the generalizability of the results to the oldest and youngest populations, complementary analysis of the oldest age group available yielded practically identical estimates and the same inferences as for the total population.

While the multiple-group ITS design is considered the strongest evaluation design when it comes to controlling for threat against internal validity, and the individual-level data enabled us to control for multiple demographic confounders with high precision, the risk for bias is not eliminated. The ITS design relies on the counterfactual assumptions of projected trends and absence of competing intervention [55], assumptions that are difficult to ascertain for large-scale reforms and dynamic populations. Moreover, even though the introduction of the reform was temporally distinct, it is possible that the impact on (inequities in) ACSC hospitalizations is delayed. Many specific aspects of PHC performance may have an impact on the rate of ACSC hospitalizations [28, 30–32], which are not empirically disentangled by the present study, but are discussed above. In addition, the outcome measure was binary which may have underestimated the true occurrence of ACSC episodes.

The exposure contrast was based on changes in degree of privatization by groups of regions, which is a large geographical area to consider. Even though this makes sense as the main responsibility for healthcare, including the implementation of the reform under study, is on the regional level, it is also a crude proxy for the local and individual-level access to primary care. Moreover, as discussed above, as the new health care centers following the reform were almost exclusively private, it is methodologically impossible to differentiate between increases in health care centers in general and increases in private health care centers. More precise exposure contrast, based on individual or small-area data would give greater precision, but this data was not available for the study, albeit urbanicity by municipality was only adjusted for in the analyses. Moreover, the region-based exposure-based contrast means that contextual rather than compositional regional level confounders are difficult to address methodologically. For example, certain regions started patient choice models earlier than the other regions, which we therefore controlled for in the adjusted analysis. Another example, not controlled in the present study, are the different reimbursement systems across the study period, and between regions. Even though the scientific evidence regarding how reimbursement systems influence PHC equity is uncertain [56], this could potentially impact the findings. Additional region-specific contextual factors could also bias the findings. Taken together, while the multiple-group ITS design used in the present study is a methodologically rigorous option compared to alternative evaluation designs, there are multiple challenges ascertaining the causal impact of large-scale and complex reforms on population outcomes, many of which are not methodologically controlled in the present study.

## Conclusions

The present study contributes with unique and rigorous evidence on the complex consequences of the Swedish 2010 choice reform, as a case reflecting the overall direction of European health policy towards patient choice, marketization, and privatization of PHC services. Results suggest that the increase in private health care centers that the reform enabled has contributed to a small improvement when it comes to overall PHC performance. At the same time, the reform was followed by increased inequities in PHC performance, with worse development in regions with large increases in private PHC provision. This is potentially rooted in the reform’s reliance on market forces as an instrument for increasing PHC provision, rather than according to the needs of the population. For the PHC system to also act as an instrument contributing towards the overall public health goal of equity in health, as is set out in Swedish legislation and policy, additional policy action is needed. Taken together, the study illustrates a potential population average vs. equity trade-off in the impact of the choice reform. This duality in the impact of the Swedish reform reflects the arguments in the European health policy debate on patient choice PHC models, with hopes of improved performance but fears of increased inequities.

## Data Availability

The dataset analysed during the current study is not publicly available due to restrictions when it comes to sharing sensitive personal data, imposed by the General Data Protection Regulation. Umeå University is the data controller. All data originates from and are conditionally available from the register holders, The Swedish National Board of Health and Welfare (https://www.socialstyrelsen.se/en/) and Statistics Sweden (https://www.scb.se/en/).
